# Iterative Development of an AI-Assisted Data Extraction Tool for Literature Synthesis in Oncology

**DOI:** 10.7759/cureus.114999

**Published:** 2026-08-22

**Authors:** Anne Liu, Faisal Alfadli, Philip Wong

**Affiliations:** 1 Radiation Medicine Program, Princess Margaret Cancer Centre, University Health Network, Toronto, CAN; 2 Faculty of Health Sciences, Queen's University, Kingston, CAN; 3 Temerty Faculty of Medicine, University of Toronto, Toronto, CAN; 4 Department of Radiation Oncology, University of Toronto, Toronto, CAN

**Keywords:** artificial intelligence in healthcare, data extraction, large language models, literature screening, prompt engineering, radiation oncology, small-cell lung cancer

## Abstract

As oncology research continues to advance, the synthesis of clinical trial evidence is becoming more demanding for healthcare professionals, yet it remains critical for improving the provision of care. While sometimes inconsistent, large language models (LLMs) have emerged as a potential solution to automate literature screening and data extraction. This technical report describes the iterative development of an AI-assisted literature synthesis tool for radiation oncology research. A screening and data extraction tool was developed in three phases using ChatGPT-4o. In Phase 1, prompts were iteratively refined to screen 840 abstracts for Phase 2/3 radiotherapy trials in small cell lung cancer (SCLC). Screening performance was compared against manual reviewer consensus using sensitivity and specificity. In Phase 2, 13 clinical trial variables were extracted from 18 eligible studies and evaluated against a manual extraction reference with a weighted scoring rubric. In Phase 3, a web-based application was developed using the optimized prompts from the first two phases and pilot-tested on eight healthcare professionals for usability and workflow relevance. In the first two phases, the initial prompts had many false positives and inconsistent extractions. Iterative prompt refinement across the second and third batches of studies improved both screening accuracy and extraction consistency. In the validation batch, the final screening model achieved 100% sensitivity and specificity, and the data extraction prompts obtained a mean score of 12.0 (SD: 0.5) out of 13. During Phase 3, pilot testers reported that the application helped them read and compare clinical trial data through concise, structured tables. Users also provided suggestions for future development, including role-dependent personalization and visualization tools. Prompt-engineered LLM models show potential for improving efficiency and accessibility of literature screening and data extraction in oncology research. Future work will integrate the feedback obtained and externally validate the tool using larger independent datasets and multidisciplinary user cohorts.

## Introduction

Clinical trials provide healthcare professionals with the most impactful results to guide their provision of care. However, as oncology research continues to expand, emerging data and evidence can outpace the capacity of providers to synthesize and interpret relevant findings. Traditional approaches to reviewing literature are often both time- and labour-intensive, which can introduce errors and biases from rapid review [1,2]. Prioritizing literature exclusively from high-impact journals risks introducing systematic publication bias. Because these prominent journals frequently favour statistically significant positive outcomes and research originating from well-funded, high-income nations, a review based solely on them may offer a skewed representation of the global body of evidence [3,4].

Recent advances in artificial intelligence (AI), specifically large language models (LLMs), have introduced new opportunities to automate parts of the literature review process [5,6]. Most notably, LLMs have demonstrated an ability to interpret unstructured text, which can vary between articles [7-10]; however, their clinical performance is inconsistent [11], and tools for data extraction have not been adequately validated for relevance or potential for workflow integration in hospital settings.

This technical report describes the pilot development and iterative optimization of an AI-assisted system for literature screening and structured data extraction in radiation oncology. Rather than evaluating a finalized AI-based system, the distinguishing contribution of this work was its iterative development framework, in which prompt-engineered workflows were progressively refined using quantitative performance measures and usability feedback. The project progressed through three sequential phases involving prompt refinement for study screening, structured data extraction, and pilot application development evaluated by healthcare professionals. Each phase was iteratively repeated and refined. After each batch within a phase was completed, qualitative observations and quantitative performance were reviewed to identify the limitations of the prompt or interface. Modifications were then implemented before the subsequent batch was tested and evaluated. This process continued for each phase until the improvements plateaued, and no major recurring issues were identified.

## Technical report

**Figure 1:**
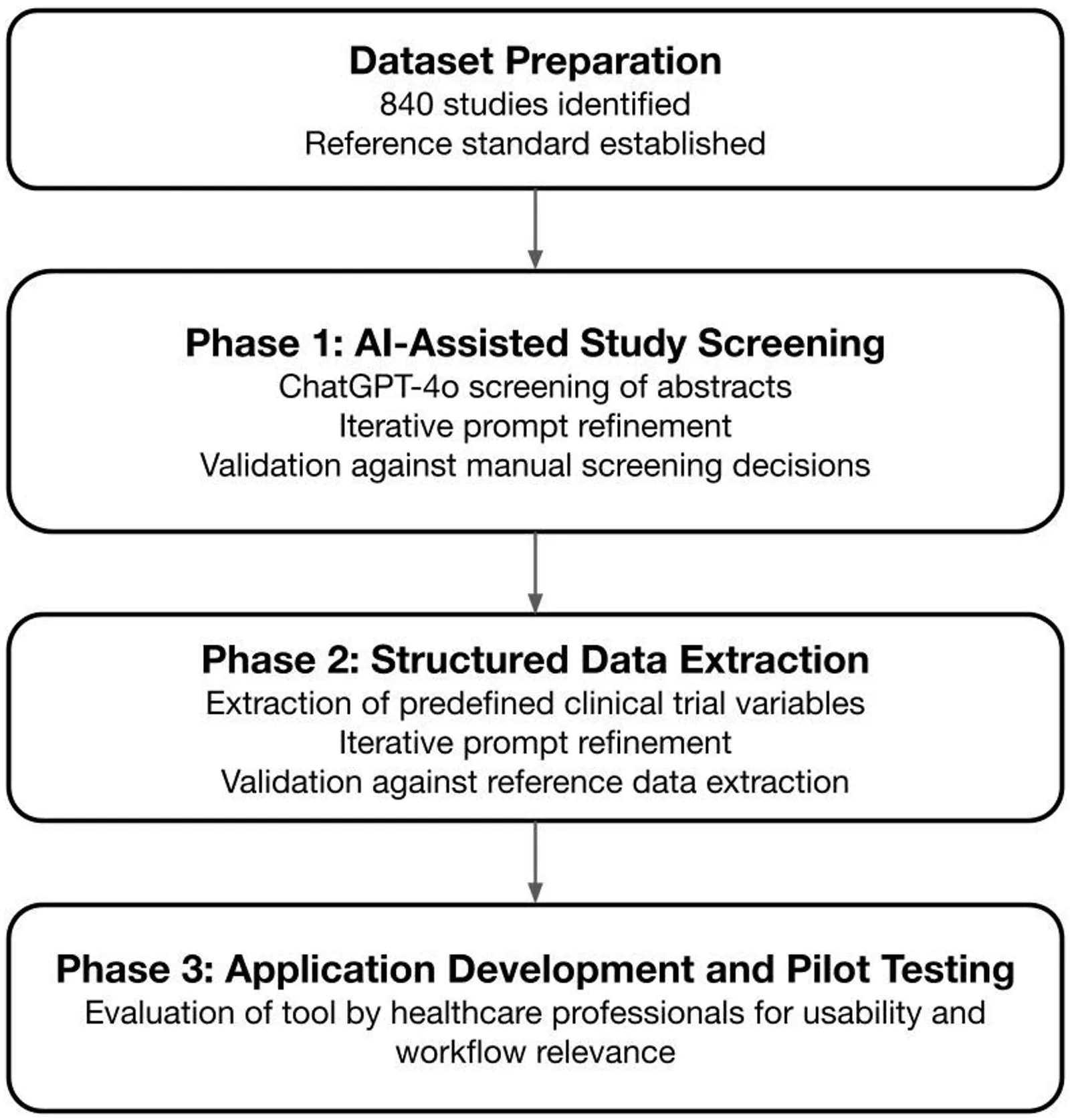
Iterative development framework for the AI-assisted literature synthesis tool After the dataset and manual reference standard were prepared, the tool was developed through three sequential phases: AI-assisted study screening, structured data extraction, and application development with pilot usability testing. Within each phase, quantitative or qualitative evaluation was used to identify limitations for refinement before progression to the subsequent phase.

The overall development and evaluation process consisted of three sequential phases that progressively refined the AI-assisted literature synthesis tool. Figure 1 provides an overview of the three-phase development and evaluation process used to create the AI-assisted literature synthesis tool.

Phase 1: The development of a study screening system 

Objective

To iteratively develop a prompt capable of accurately screening oncology clinical trials for literature review. 

Methods

The initial phase of the project focused on developing a prompt-engineering-based system to identify relevant trials in radiation therapy for small cell lung cancer (SCLC). The inclusion criteria focused on Phase 2 or 3 clinical trials reporting radiation therapy interventions and outcomes related to survival, tumour response, and safety. To determine the capacity of an LLM to screen studies, a literature search was performed using MEDLINE and Embase to identify studies evaluating radiation therapy in SCLC. After duplicates were removed, 840 abstracts were available for title and abstract screening. All articles were manually screened by at least two independent reviewers, and conflicts that arose were discussed by the reviewers to reach consensus. Of the 840 studies, 18 met all the inclusion criteria during manual screening. A prompt was then developed to instruct ChatGPT-4o to screen articles based on the identified inclusion criteria. The prompt also instructed the LLM to report its confidence in each inclusion or exclusion decision based on predefined criteria related to the level of detail available in the abstract. Five study abstracts were provided as input at a time, as this was the maximum number of studies that did not appear to decrease output quality. The prompt for each batch of studies was entered independently in a separate ChatGPT-4o conversation, which ensured that context from previous conversations was not carried over to subsequent batches, thereby confirming that the outcomes were purely based on the prompt.

Results

The 840 abstracts were randomly divided into three batches of 280 studies. ChatGPT-4o was instructed to screen the first group of study abstracts, which contained 10 articles that were manually included. The screening outputs for the first batch showed that the LLM included several studies unrelated to SCLC or not focused on radiation therapy, both of which were primary inclusion criteria. Overall, the AI output had 21 disagreements with the manual results, with a sensitivity of 100% and a specificity of 94.3%. Six articles were also classified as neither included nor excluded and were instead marked "neutral," suggesting that the inclusion criteria were not written clearly enough into the prompt to ensure full confidence in its decisions.

The prompt was refined twice more and applied to the second and third datasets after each respective revision. These refinements primarily focused on simplifying the structure of the prompt and making the inclusion criteria stricter to improve specificity. For example, by explicitly excluding terms indicating non-SCLC, rather than solely including articles focused on SCLC, the specificity increased to 97.8%, suggesting that the strictness of the criteria helped exclude false positives. Concurrently, sensitivity in the second batch decreased to 83.3%, suggesting that the criteria may have been written too strictly to identify all eligible studies accurately, thus increasing false negatives. The final round of refinements for the third group of data aimed to create a balance between the first two large-scale refinements and introduced strict checks to ensure consistency between the inclusion or exclusion decisions and the reported confidence level. Specifically, the prompt was refined to remove redundancy, reduce overall length, and reorder the instructions according to their importance to ensure that the criteria were properly followed. In the final group, the sensitivity and specificity both increased to 100% with no false positives or negatives. Table 1 shows the number of true positives, true negatives, false positives, and false negatives from each batch, alongside the associated specificity and sensitivity. The absence of neutral markers in the second and third batches suggests improved clarity of the prompt, which increased the confidence of ChatGPT-4o in its decisions.

**Table 1:** Summary of quantitative results showing the number of true positives (TP), true negatives (TN), false positives (FP), and false negatives (FN), along with the derived specificity and sensitivity values, from the screening tool

Dataset	TP	TN	FP	FN	Neutral	Specificity	Sensitivity
Batch 1	10	249	15	0	6	94.3%	100%
Batch 2	5	268	6	1	0	97.8%	83.3%
Batch 3	2	278	0	0	0	100%	100%
Final Evaluation Batch	6	274	0	0	0	100%	100%

Because the final development batch contained only two eligible studies, the finalized prompt was additionally evaluated using a randomly selected 280-abstract resampling of the original dataset. This analysis represented internal retesting rather than independent external validation because the included abstracts could overlap with those used during prompt development. As in the third batch, the prompt was not changed for this final batch, and the resulting specificity and sensitivity were 100%. At this stage, all included studies met the predefined criteria based on abstract review.

Phase 2: The development of a structured data extraction tool 

Objective

To iteratively optimize AI-assisted extraction of relevant clinical trial variables and maintain consistency across repeated outputs. 

Methods

Following the optimization of article screening, the project was expanded to support the next stage of literature synthesis: structured data extraction. A pilot set of the 18 studies identified during Phase 1 was used for this second phase of development.

The variables selected to be extracted were obtained from content typically gathered when reading through radiotherapy clinical trials. A total of 13 variables were selected for extraction, all of which fell into one of five categories: study metadata, population characteristics, interventions, outcomes, and conclusions. As in Phase 1, a reference extraction was first created using the variables manually extracted by two independent reviewers who reached consensus when conflicts arose. The 18 studies were randomized into three groups of six, and a prompt was designed to extract all 13 variables from the abstract of each entered study. Each study was independently submitted using the prompt in 10 separate conversations, producing 10 independent extractions for each study. This meant that, in each group of six studies, a total of 60 chats would be created and analyzed. The purpose of including repeated extractions was to determine the consistency of outputs across independent conversations. The prompt was continuously refined with each study batch, and the results of the data extracted by AI were compared to the reference extraction.

For the final statistical analysis, each variable extracted by ChatGPT-4o was scored independently using a three-level weighted rubric. For the study metadata, population characteristics, interventions, and outcomes sections, the data were scored as correct (1.0 point) if fully accurate and appropriately categorized; partially correct (0.5 points) if factually correct but incomplete, miscategorized, or redundant; and incorrect (0.0 points) if factually inaccurate or omitted despite being reported in the original abstract. The three-level rubric was selected as a development metric to distinguish fully correct extractions from outputs that retained factually correct information but contained incomplete or structural errors; it was not intended as a previously validated psychometric or clinical measurement scale. The average score of each variable within a single study was calculated using the mean of the 10 scores reported for that variable, corresponding to the 10 outputs of each study. The average score of each study was calculated as the mean of the 13 variable scores.

Results

The development of the structured data extraction tool relied on a hybrid approach of initial qualitative optimization followed by a final quantitative evaluation. During the first batch of studies, the initial prompt resulted in substantial inclusion of extraneous information, and the extracted variables were inconsistently categorized. Given the predefined extraction requirements, extracted information was often either overly brief or excessively detailed, which could make simply reading the original abstract more efficient in practice. For example, in the first stage, studies containing both retrospective and prospective components were not properly separated, so retrospective data were included even when only prospective trial data met the extraction criteria. Additionally, treatment arms were often merged, limiting outcome comparisons across groups based on treatment dose or intervention schedule.

In the second batch, these structural issues were resolved qualitatively through manual prompt refinement; however, formatting and wording consistency issues remained prevalent, limiting the ability to easily understand study data. Because these first two batches were assessed through iterative, qualitative error analysis to rapidly refine the prompt parameters, formal quantitative scoring was reserved for the final evaluation stage. With further refinement of variable definitions and tabular output standardization, the consistency and interpretability of the results improved, and hallucinations of non-existent details decreased.

Across the final six evaluated studies, the mean extraction score was 12.0 (SD: 0.5) out of 13 points. Mean accuracy scores for each variable are summarized in Table 2.

**Table 2:** Mean accuracy of the extracted variables from the final six evaluated studies

Variable	Mean Accuracy
Study design	0.82
Trial registry number	0.80
Disease indication	1.00
Inclusion criteria and patient characteristics	0.88
Intervention	0.92
Enrollment	0.92
Response rate	1.00
Rate of progression	0.99
Survival	0.88
Extraneous endpoints	0.89
Adverse events and toxicities	1.00
Follow-up duration and trial location	0.92
Conclusion	1.00

The disease indication, response rate, adverse events and toxicities, and conclusions achieved perfect mean accuracy scores (1.00), while the trial registry number had the lowest mean accuracy (0.80).

Phase 3: Application development and clinical testing 

Objective

To develop and pilot-test a user application implementing the optimized extraction workflow for automated literature interpretation and comparison.

Methods

**Figure 2:**
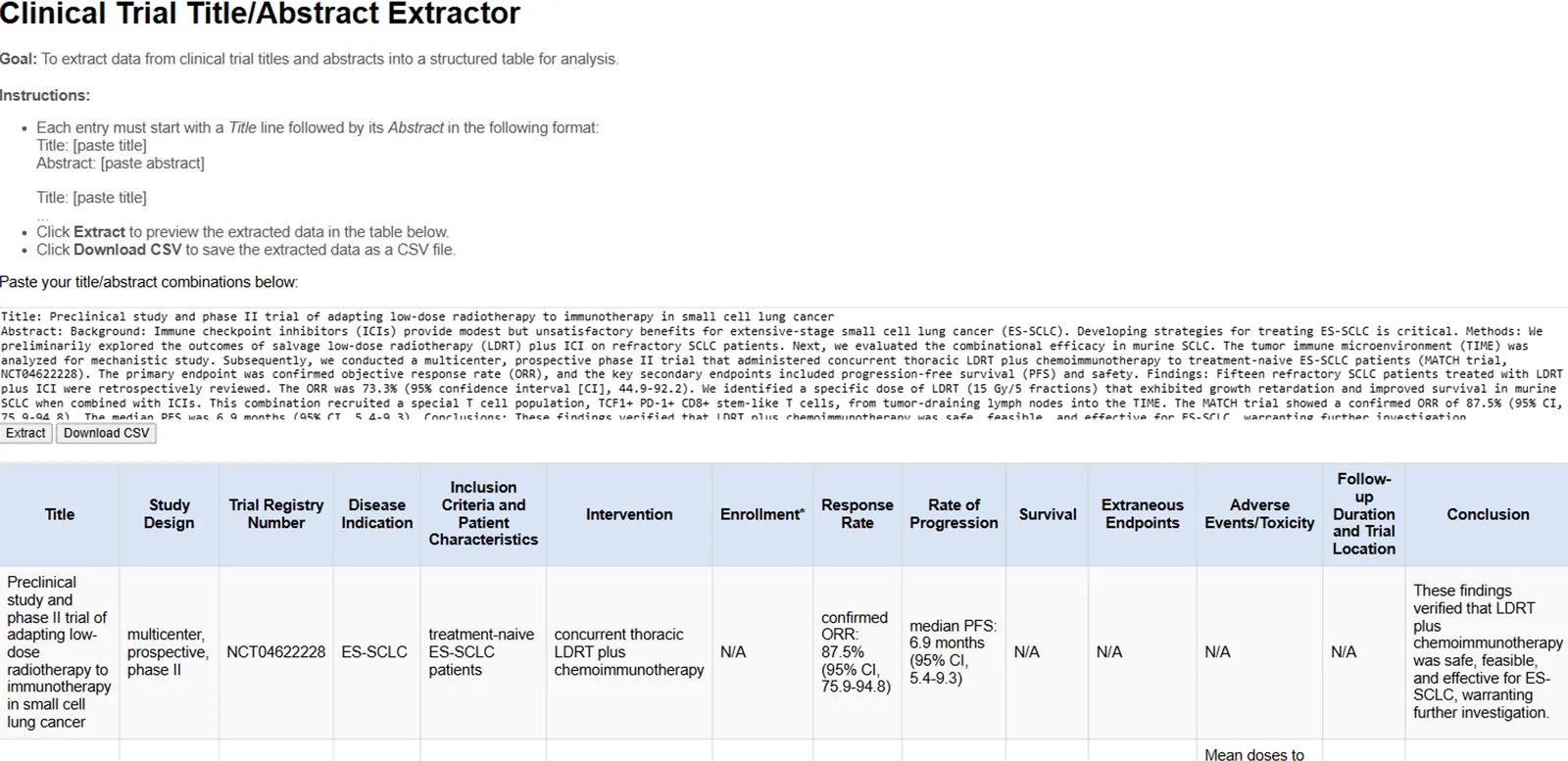
User interface of the AI-assisted literature synthesis application demonstrating automated extraction of the predefined clinical trial variables into a standardized comparison table

Due to the experimentally determined limitations of the ChatGPT-4o interface, only five articles could be entered for screening at a time during the first phase. When this number was exceeded, the tool began to deviate from the instructions provided, generating incorrectly formatted tables, incomplete information, more frequent errors, or hallucinated content. To address this usability limitation, a web-based application was developed to use the previously developed extraction workflow with an API, allowing users to input multiple clinical trial abstracts and generate formatted tabular outputs containing the predefined variables. Figure 2 demonstrates the data extraction process using a representative study included in the evaluation dataset [12].

The pilot website was evaluated by eight healthcare professionals, including radiation oncologists, researchers, and clinical trial nurses, of whom seven completed the post-test feedback survey. Prior to completing a feedback survey, participants completed an assessment that involved reviewing two groups of five clinical trial abstracts each. One group was reviewed manually and the other using the AI data extraction tool. After completing both tasks, participants completed surveys evaluating the ease of use and clarity of presentation of the tool, their overall satisfaction, and the potential for integration into existing literature review workflows. This comparison was particularly relevant because manual interpretation was reported as the conventional alternative to using the application. Clinical usability was defined as the perceived ease with which participants could use the application to quickly interpret and compare clinical trial findings within their existing literature review workflow. Users also provided qualitative feedback to support further development and personalization of the application for different healthcare professionals.

In addition to usability testing, physician participants assessed whether the extracted study characteristics accurately reflected the information presented in the original abstracts and identified any important omissions or inaccuracies in the feedback form. These observations served as an additional quality assurance step to improve subsequent refinements of the prompt structure and the user interface of the application.

Results

From the feedback survey, free-text responses pointed to three recurring themes. First, participants consistently perceived the application as reducing the time required to review and compare clinical trials by summarizing long abstracts into structured tables. As one resident commented, the tool "quickly differentiates relevant vs irrelevant trials by displaying information in a clean, simplified table." Second, several participants requested that extracted variables be customized and visualized in different ways, including through graphed comparisons of survival outcomes and the ability to select relevant variables. Finally, though participants generally considered the extracted information accurate, several noted that they would initially verify the AI-generated outputs against the original abstracts before consistently relying on the application.

Overall, the results of the pilot project suggest that the web-based application may improve efficiency and accessibility in clinical data extraction workflows, as it was generally perceived as accurate and easy to use. Based on the feedback received, further development is ongoing to improve interface design and options for the extracted data.

## Discussion

This technical report describes the pilot development and iterative optimization of an AI-assisted pipeline to complete literature screening and data extraction in radiation oncology, demonstrating both the potential and limitations of applying LLMs to clinical tasks requiring precision. The findings show the importance of iterative refinement in improving the accuracy of LLM output in prompt engineering tasks, aligning closely with studies conducted on AI use in healthcare [13]. Although the included studies focused on radiation oncology, the broad nature of the variables extracted, including study design and overall survival, suggests that this tool may be feasible in other clinical contexts.

Recent studies have shown that LLMs can perform a range of tasks related to research and clinical care, such as summarizing complex health information for patient understanding, answering questions about healthcare, and data extraction in electronic health records [14-16]. Performance in these contexts has been variable, and to optimize accuracy, many existing automated systems have primarily relied on machine learning approaches to screen abstracts and classify summarized data with supervised models on labelled datasets [17-19]. These models have shown high efficiency and accuracy but have notable limitations related to their need for curated training data and limited generalizability across fields. A prompt-engineering-based technique, such as the pipeline described in this study, may improve the flexibility of AI-based literature synthesis while reducing reliance on curated training datasets. Still, the use of LLMs is often associated with low specificity and sensitivity, particularly when unrefined prompts are used for articles with nuanced differences in study design or clinical context.

During the first two phases involving prompt engineering, a key observation was the need to balance specificity and sensitivity in designing the prompt. In both phases, the initial prompts were overly open and permissive, which resulted in high false-positive rates and limited practical utility; users would still have needed to manually identify relevant studies or locate key data within overly detailed descriptions provided under each variable. The subsequent refinements in the second stages of each phase improved specificity but reduced sensitivity by excluding eligible articles. Through the process of iterative refinement, a recommended practice in prompt engineering, a balance was adequately reached between sensitivity and specificity to optimize the model.

For the second phase, it was important to consider the challenges regarding information categorization and presentation, as these ultimately represent the outcomes evaluated by healthcare professionals. The tool will be continuously developed to optimize the presentation of identified variables and integrate the feedback received, such as generating graphs or clear data tables comparing specific variables across different studies, particularly because the pilot evaluation showed that participants differed in the information they sought to extract from clinical trials. Some users preferred concise summaries that focused on clinical outcomes, while others required greater methodological detail or comparisons between treatment arms. This suggests that future AI-assisted literature synthesis tools should prioritize customizable outputs rather than a single structured summary.

Additionally, the performance of both models, particularly the extraction model, was observed to be dependent on the size of the input, defined as the number of articles included in a single input. The quality of the output was observed to decline when more articles were processed simultaneously. This was the main driver for the development of the website that could automate continuous processing of articles individually on the back end. The trend of decreasing quality is consistent with prior research describing the attention limitations of LLMs, which have been shown to affect performance in tasks requiring multiple tests [20].

Current limitations of the tools described include the small sample size for data extraction of 18 studies, the small sample size of eight healthcare professionals in the usability evaluation, and the narrow focus of articles on radiation therapy in SCLC, which limit the generalizability of the observed extraction performance and usability findings. In addition, the finalized screening prompt was internally retested using a resampled subset of the original dataset rather than an independent external dataset, and external validation will therefore be required to determine whether its performance generalizes to other literature corpora and clinical domains. The pilot usability evaluation also only assessed a single interaction with the application and thus could not assess whether users changed their literature review strategy or efficiency after repeated exposure to the tool. Though this project primarily served as a pilot study, future work should examine how the adoption of this tool can affect strategies for literature review over time. Additionally, the first two phases relied exclusively on information presented in the abstracts, as this information often highlights the most important details in the full-length article.

Overall, the findings are consistent with recent work in the clinical use of AI, suggesting that automated screening and data extraction can support more efficient evidence review. From the feedback received from healthcare professionals, the usability and workflow integration capacity of the tool will continue to be improved and tested in larger populations.

## Conclusions

This pilot project demonstrates the feasibility of iteratively developing and refining an AI-assisted literature synthesis tool through prompt engineering and continuous quantitative validation combined with user feedback. Future work will focus on external validation using larger independent datasets and multidisciplinary user cohorts, increasing customization options for different healthcare professionals, and evaluating the long-term effects of the tool on literature review efficiency and data extraction strategies.
